# Systematic review and meta-analysis of the prognostic value of preoperative platelet-to-lymphocyte ratio in patients with urothelial carcinoma

**DOI:** 10.18632/oncotarget.21162

**Published:** 2017-09-22

**Authors:** Shuiqing Wu, Qi Wan, Ran Xu, Xuan Zhu, Haiqing He, Xiaokun Zhao

**Affiliations:** ^1^ Department of Urology, The Second Xiangya Hospital, Central South University, Changsha, Hunan Province, People’s Republic of China; ^2^ MRC Centre for Reproductive Health, Queen’s Medical Research Institute, Edinburgh, United Kingdom; ^3^ Department of Neurology, The First Hospital of Changsha, Changsha, Hunan Province, People’s Republic of China

**Keywords:** platelet-to-lymphocyte ratio, urothelial carcinoma, prognostic factor, meta-analysis

## Abstract

A large number of studies have investigated the prognostic value of the platelet-to-lymphocyte ratio (PLR) in patients diagnosed with urothelial carcinoma, but the evidence from these papers is conflicting. This systematic review and meta-analysis was carried out to assess the role of PLR in urothelial carcinoma patients. After a systematic search of the PubMed, Embase, Web of science databases, the associations between PLR and overall survival (OS), cancer-specific survival (CSS)/disease-specific survival (DSS), and relapse-free survival (RFS)/disease-free survival (DFS) were analyzed in urothelial carcinoma patients. The relationship between PLR and pathological results was also evaluated. A total of seven studies (eight cohorts) comprising 3171 patients were included. The pooled hazard ratios (HRs) and 95% confidence intervals (CIs) indicated the increased preoperative PLR predicted poor OS (HR = 1.14, 95% CI = 1.01- 1.27, *p* < 0.001), CSS/DSS (HR = 1.24, 95% CI = 1.08–1.40, *p* < 0.001), RFS/DFS (HR = 1.23, 95% CI = 1.09–1.38, *p* < 0.001). However, no significant correlation was found between elevated preoperative PLR and pathological results such as tumor grade, tumor necrosis and T stages. These findings suggest a high PLR is associated with reduced OS, CSS/DSS and RFS/DFS in urothelial carcinoma. Preoperative PLR may therefore be a predictive factor in this patient group.

## INTRODUCTION

Urothelial carcinomas arise throughout the length of the urinary tract, though most develop in the lower urinary tract and are termed lower urinary tract urothelial carcinomas (LUTUC). Upper urinary tract urothelial carcinomas (UUTUC) are relatively rare, accounting for less than 10%. Urothelial carcinoma of the bladder (UCB) is the main type of LUTUC. It is a common urinary malignancy [1], contributing to 7% of all new cancer diagnoses and 4% of all cancer mortality. The gold standard for treating muscle invasive UCB is radical cystectomy, while the recommended treatment for UUTUC is usually radical nephroureterectomy (RNU) with bladder cuff excision (BCE). Kidney-sparing treatment using endoscopic approaches have also been recommended for low-grade and low-stage UUTUC [2, 3]. At present, conventional tumor grading and pathological staging are the best way to inform prognosis. However, an optimal method of pre-operative tumor staging is still debated, as imaging and local biopsy are insufficient for this purpose [3–5]. This makes it important to validate proposed preoperative prognostic biomarkers, as these may be able to improve risk stratification and clinical decision making for patients with urothelial carcinomas.

Recent evidence suggests the immune system plays an essential role in the initiation, development and progression of urothelial carcinomas [6, 7]. Some studies suggest platelets are associated with the formation of early metastatic niches, and enhance the metastatic potential of cancer cells [8, 9]. Lymphocytes are also key determinants of the prognosis of urothelial carcinomas [10, 11]. With circulating blood biomarkers showing the potential to serve as cost-effective prognostic predictors in cancer patients [12], the platelet-to-lymphocyte ratio (PLR) has been investigated in various cancer types, with an elevated PLR associated with poor survival [13–14]. The PLR has also been evaluated as a possible prognostic marker for urothelial carcinomas; however, the prognostic value of an elevated PLR in urothelial carcinomas remains uncertain due to limited sample sizes and variation within single studies [15–21]. We therefore performed this systematic review and meta-analysis to assess the relationship between prognostic variables and the PLR derived from preoperative blood samples collected from urothelial carcinoma patients.

## RESULTS

### Search results and characteristics of included studies

**Figure 1:**
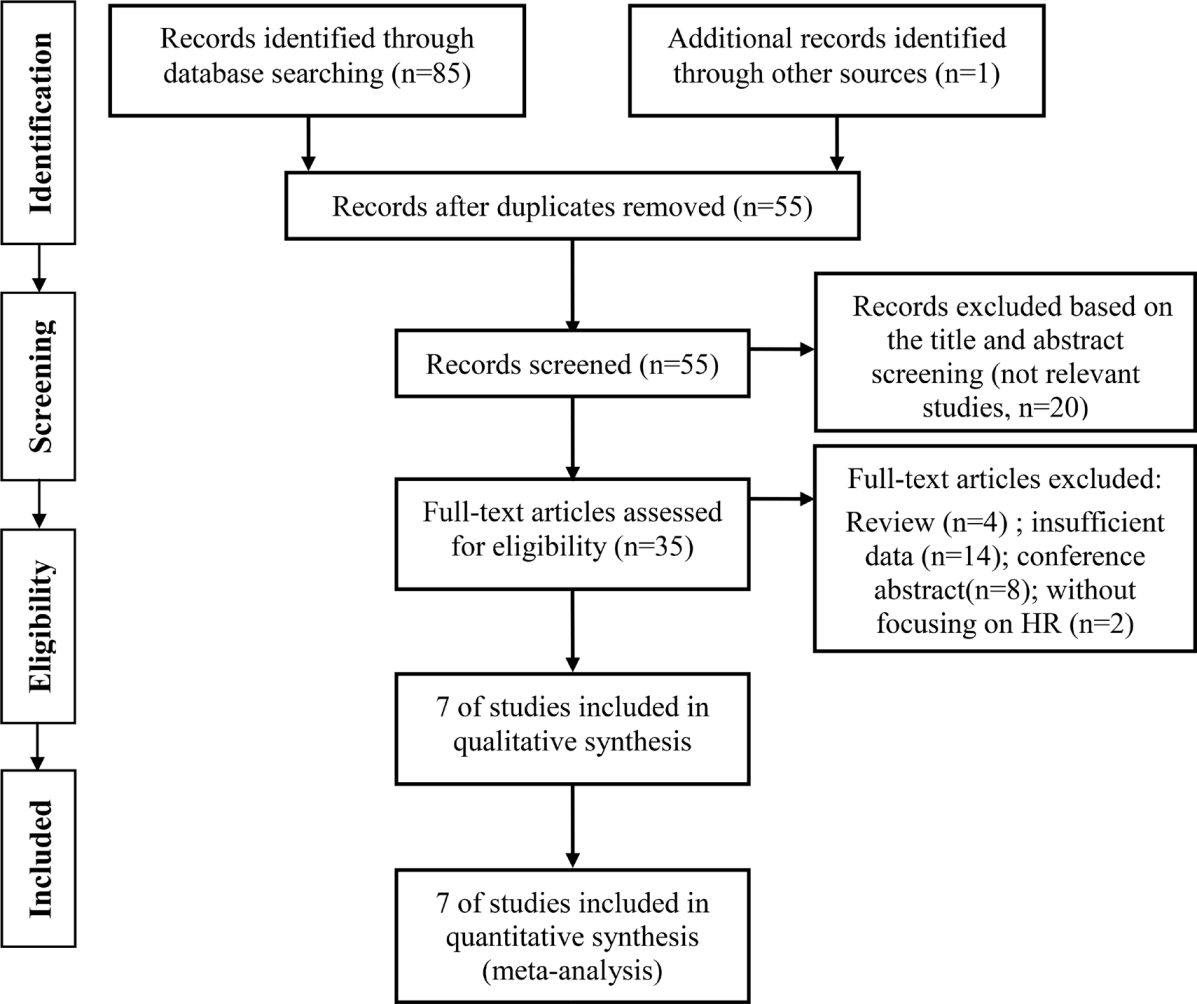
Flow diagram for the selection of included articles

85 relevant studies were initially identified from the databases and reference lists using our search strategy (described in detail in the materials and methods section). 55 studies remained after the deletion of duplicates detected by Mendeley software and double checked manually. 20 further studies were excluded for not relevant with prognostic analysis after screening of the titles and abstracts. The rest of the studies were fully assessed, and only 7 full text studies comprising 3171 patients met our inclusion and exclusion criteria, again detailed in the materials and methods section [15–21]. The PRISMA flow diagram describing theliterature search was shown in Figure 1. The included studies were published between 2015 and 2017. All the studies were published in English. 6 of 7 the studies were retrospective, with the remaining one study not reporting its design pattern [15]. The sample sizes in the included studies ranged from 124 to 1551. Overall survival (OS), cancer specific survival/disease specific survival(CSS/DSS), relapse-free survival/disease-free survival (RFS/DFS) were investigated together or separately as prognostic endpoints in the included studies. The cut-off values of PLR in the studies ranged from 124 to 300. We extracted the hazard ratios (HR) and 95% confidence intervals (CI) directly from the included studies. For Kim’s study, we extracted two HRs and 95% CIs based on the two cohorts, focusing on different populations in the study with different cut-offs [15]. The prognostic role of PLR in pathological results was investigated in 3 studies, and pathological T stage, tumor grade and tumor necrosis were included as main pathological results. The detailed characteristics of included studies were shown in Table 1. As for the quality assessment of the included studies, The global quality score ranged 63.6–72.7%, with a median of 67.2%. The laboratory methodology subscore had the lowest value, with a median value of 5.7 out of 14, and the most poorly described items were tissue sample conservation, blinding assessment, definition of the level of positivity of the test. The details of quality assessment was shown in Table 3.

**Table 1:** Characteristics of the included acticles

First author (year)	Design	Area	Cases number	types	T Stage	therapy	Cut-off value	Endpoints	HR (95% CI)
Kim (2015)	NR	Korea	277	UUTUC	Ta,Tis,T1-T4	RNU	150^a^;150-300;300	DFS,DSS	U
Zhang (2015)	Retrospective	China	124	UCB	T1-T4	RC	140	OS	M
Bhindi (2016)	Retrospective	Canada	418	UCB	Ta,Tis,T1-T4	RC	per 100 units	RFS,CSS,OS	M
Huang (2016)	Retrospective	China	481	UUTUC	Ta,T1-T4	RN	241.2	OS,CSS	M
Kang (2017)	Retrospective	Korea	1551	UCB	Ta,Tis,T1	TURBT	124	OS,CSS	M
Song (2016)	Retrospective	China	140	UUTUC	Ta,Tis,T1-T4	RNU with BCE	128	DFS,PFS	M
Dalpiaz (2017)	Retrospective	Austria	180	UUTUC	T1-T4	RNU,SU	150	OS,CSS	M

UUTUC: upper urinary tract urothelial carcinoma; UCB: urothelial carcinoma of bladder; RFS: recurrence free survival; CSS: cancer specific survival; RNU: radical nephroureterectomy; SU: segmental ureterectomy; RC: radical cystectomy; BCE: bladder cuff excision; M: multivariate analysis; U: univariate analysis; ^a^: < 150 as reference.

**Table 2:** The association between high pretreatment PLR level and pathological characteristics

Characteristics	Studies (Ref No.)	Pooled OR (95% CI)	Heterogeneity assessment			
			Chi^2^	I^2^	*P* value	
pT stage						
T≥T2 vs. T≤T1	[16, 21]	1.87 (0.59, 3.14)	2.8	64.3%		0.094
T≥T3 vs. T≤T2	[16, 20]	1.87 (0.78 , 2.97)	1.26	20.3%		0.263
Tumor grade						
G≥3 vs. G≤2	[20, 21]	1.63 (0.81, 2.45)	0.54	0.0%		0.462
Tumor necrosis						
present vs. absent	[20, 21]	2.11 (-0.21, 4.44)	0.22	0.0%		0.641

pT stage: Pathological T stage; G: tumor grade; vs: versus; Ref: Reference number.

**Table 3:** Methodological assessments of the studies included in the meta-analysis

Author (year)	Global score (%)	Scientific design (Total score = 10)	Laboratory methodology (Total score = 14)	Generalizability (Total score = 12)	Results analysis (Total score = 8)
Kim (2015)	63.6	6	6	10	6
Zhang (2015)	65.9	8	6	10	5
Bhindi (2016)	68.2	7	4	12	7
Huang (2016)	70.5	8	6	12	5
Song (2016)	65.9	7	6	10	6
Kang (2017)	63.6	7	6	8	7
Dalpiaz (2017)	72.7	8	6	10	8

### Prognostic role of PLR in OS

**Figure 2:**
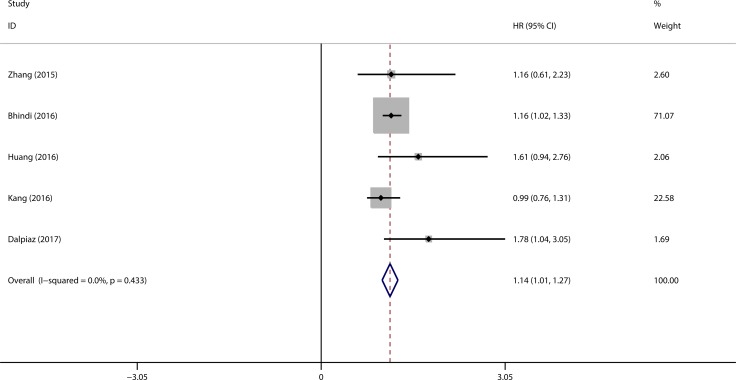
Forest plot evaluating the prognostic role of high PLR on OS

Five studies comprising 2754 patients were included in the meta-analysis of PLR in OS. The cut-off values ranged from 124 to 241.2. For zhang’s study, there are two HRs derived from the analysis with 140 used as cut-off value or continuous variable in the same population. To decrease the heterogeneity among the studies, we just extracted the HR and related 95% CI from the analysis with 140 used as cut-off value. The pooled HR from meta-analysis was 1.14 (95% CI = 1.01–1.27, *p* < 0.001) with no significant heterogeneity among the studies (I^2^ = 0.0%, P_h_ = 0.433), as shown in Figure 2. Furthermore, sub-group analyses were performed according to the cancer types, stratification of cut-off values, number of patients, ethnicity. The results from sub-group analyses were listed in Supplementary Table 1. There was no significant heterogeneity among the studies included for all the analyses.

### Meta-analysis of PLR in DSS/CSS

**Figure 3:**
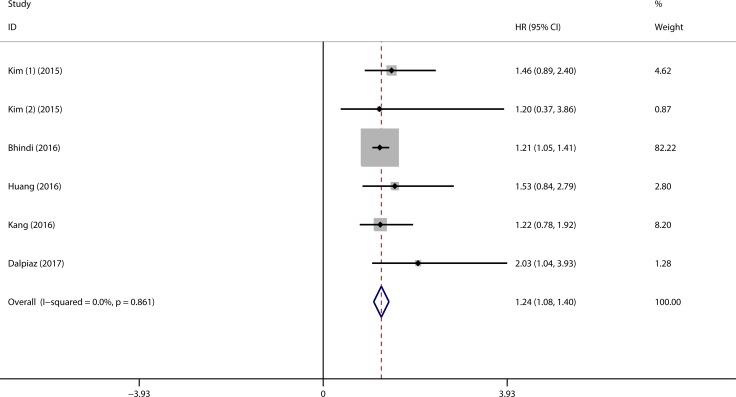
Forest plot assessing the association of high PLR on DSS/CSS (1). HR derived from cohort one (150-300 vs. < 150); (2). HR derived from cohort two (> 300 vs. < 150).

Five studies with six cohorts investigating the association between PLR and CSS/DSS were included in this system review and meta-analysis, which comprising 2907 patients in total. The cut-off values ranged from 124 to 300 in the included studies. The patients were divided into three groups according to the stratified PLR cut-offs in Kim’s study, which comprised two cohorts focusing on the prognostic function of PLR with DSS in different population. Therefore, we can extract two HRs and related 95% CIs from Kim’s study. As shown in Figure 3, the pooled HR indicated the elevated PLR was associated with worse DSS/CSS in urothelial carcinoma (HR = 1.24, 95% CI = 1.08–1.40, *p* < 0.001) (Figure 3). We performed the sub-group analysis according to the different cancer types, and the results also indicated high PLR was correlated with reduced DSS/CSS in UUTUC and UCB patients, and the pooled HRs were 1.53 (95% CI = 1.01–2.06, *p* < 0.001) and 1.21 (95% CI = 1.04–1.38, *p* < 0.001), respectively. Moreover, sub-group analyses categorized by number of patients, ethnicity, cut-off values were performed, and all the results were listed in Supplementary Table 2. No significant heterogeneity was detected among the cohorts included for all the analyses.

### Meta-analysis of PLR in RFS/DFS

**Figure 4:**
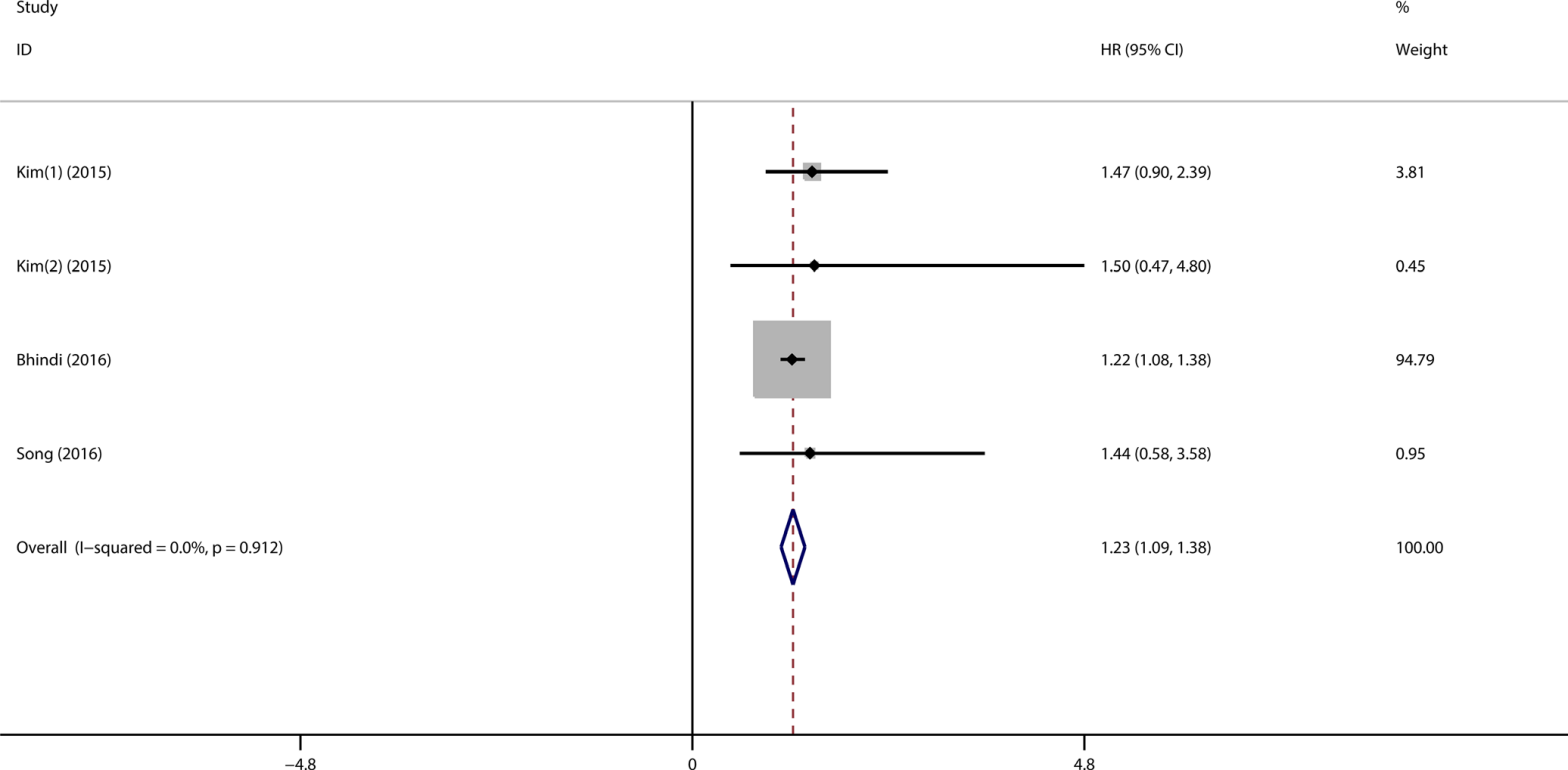
Forest plot evaluating the correlation of high PLR on RFS/DFS (1). HR derived from cohort one (150–300 vs. < 150); (2). HR derived from cohort two (> 300 vs. < 150).

Three studies comprising 835 patients investigated the prognostic role of PLR in RFS/DFS. As mentioned in previous paragraph, two HRs and related 95% CI were extracted from Kim’s study, for the reason that they were derived from different groups of patients. The results of meta-analysis suggested the patients with higher PLR had a unfavorable RFS/DFS (HR = 1.23, 95% CI = 1.09–1.38, *p* < 0.001) (Figure 4).

### Association between high PLR and pathological results

The relationship between PLR and pathological results was reported in three studies. We extracted the raw data and obtained the odds ratios (ORs) and related 95% CIs with SPSS 19.0 software. However, the pooled OR results derived from meta-analysis indicated the higher PLR was not an independent risk factor for existence of tumor necrosis, high tumor grade (G ≥ 3) and pathological T stages (T ≥ 2). The pooled ORs and 95% CIs were shown in Table 2.

### Assessment of publication bias

**Figure 5:**
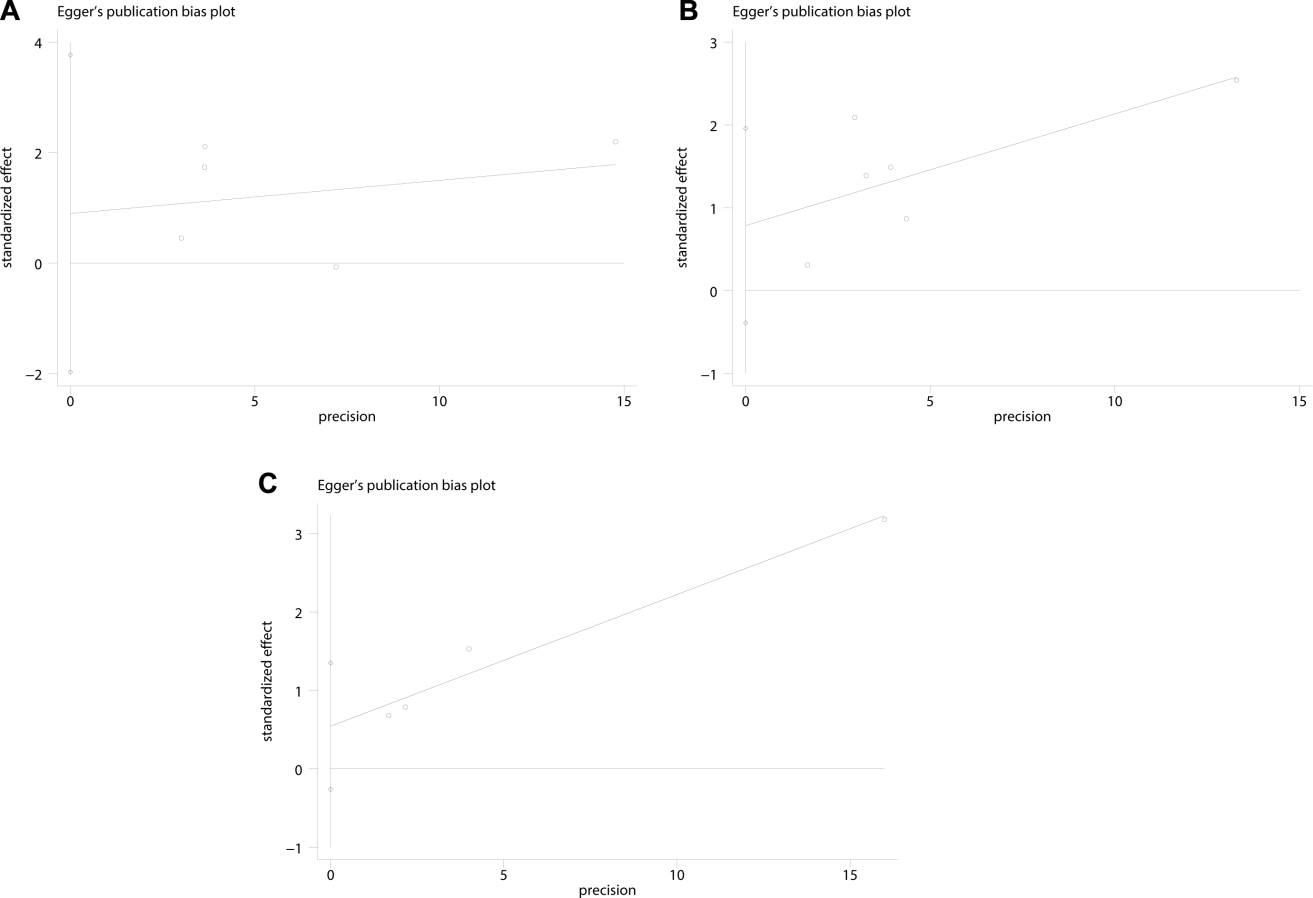
Egger’s linear regression test evaluating the potential publication bias among the included studies (**A**) PLR with OS (*P* = 0.394); (**B**). PLR with DSS/CSS (*P* = 0.137); (**C**). PLR with RFS/DFS (*P* = 0.101).

Begg’s funnel plot and Egger’s test were performed to assess the publication bias of the included studies. No significant asymmetry was found by Begg’s and Egger’s tests in the included studies concerning PLR in OS, PLR in DSS/CSS, PLR in RFS/DFS. The results from Egger’s test were shown in Figure 5. As the number of studies included less than 10, the results from Begg’s test should be interpreted cautiously (Supplementary Figure 1). Furthermore, we conducted “trim and fill method” to find and include hypothesized missing studies. Two hypothesized missing studies were found concerning PLR in OS, PLR in DSS/CSS, PLR in RFS/DFS, respectively. No significant heterogeneity was detected among the included studies and the adjusted results from fixed effect model did not influence the conclusions (Supplementary Figure 2).

## DISCUSSION

Despite developments in urothelial carcinoma treatment, improving long-term survival remains a major challenge [16, 22]. Recent studies have investigated molecular predictors that may facilitate prognostication and individualized therapy [23, 24]. Cells and molecules associated with the inflammatory response play an essential role in tumor initiation, progression and invasion [19, 25], and have therefore been of particular interest. The interaction between cancer and immune cells can be reflected by hematological and biochemical parameters. Previous studies have reported the prognostic value of inflammatory indicators such as C-reactive protein, macrophages, and PLR [26–28]. However, only limited conclusions can be drawn from the individual studies due to their small sample sizes and population heterogeneity. This makes it essential to reliably assess and validate the various possible risk factors.

Pre-operative PLR – the absolute platelet number divided by the lymphocyte count before surgery – has been investigated as an independent predictor in urothelial carcinoma patients [15–21, 28]. In this systematic review and meta-analysis, we assessed prognostic value of the PLR in urothelial carcinomas. Seven studies with eight cohorts comprising 3171 urothelial carcinoma patients were included in our analysis. The pooled HRs indicated that a high preoperative PLR was associated with reduced OS, CSS/DSS, and RFS/DFS in urothelial carcinoma patients, and no significant heterogeneity was found among the studies.

The included urothelial carcinoma patients can be divided into those with bladder cancers and those with upper urinary tract cancers. We then performed a subgroup analysis to evaluate the role of an elevated PLR in urothelial carcinomas classified with two types based on their different anatomical locations (UUTUC, UCB). The pooled HRs derived from the sub-analysis suggested an elevated PLR was associated with poor OS and CSS/DSS in UUTUC patients. For the UCB patients, however, the high PLR predicted poor CSS/DSS, while no significant correlation was detected between a high PLR and OS. We also performed subgroup analyses categorized by the number of patients (> 400 and < 400), cut-off values (≥ 150, < 150 and Per 100 unit), and ethnicity (Asian and Caucasion). The pooled results should be cautiously interpreted due to the limited number of studies included for meta-analysis. Additionally, we used raw data from the included studies to calculate ORs to analyze the relationship between the PLR and selected pathological parameters [16, 20, 21]. The pooled ORs revealed that a high PLR was not an independent risk factor for advanced pathological tumor stage (T ≥ 2), the presence of tumor necrosis or high tumor grade (G ≥ 3). Although there is no significant heterogeneity among the studies included for sub-analysis, the limited number of patients mean that more related studies will be required to validate the conclusions drawn from this review and meta-analysis.

As far as we know, this is the first systematic review and meta-analysis to evaluate the prognostic value of PLR in urothelial carcinomas. This study is not without limitations. First, most of the included studies were retrospective, which may contribute to the heterogeneity in patient selection. Second, the studies included for OS, CSS/DSS and RFS/DFS analyses were relatively limited, and the results derived from subgroup analyses should be interpreted cautiously. Third, some studies focusing on the prognostic value of PLR in urothelial carcinomas were excluded because the data that could be extracted for HRs and 95% CIs was insufficient [27, 28], which may have influenced the interpretation of our study. Fourth, nearly all the included studies presented multivariate HRs and 95% CIs with consideration of other confounding factors for the meta-analysis. However, one study with only a univariate HR result was also included in the pooled analysis [15], which might have led to the potential heterogeneity among the studies. Despite this, on the whole no significant heterogeneity was detected among the included studies.

Collectively, the findings of our systematic review and meta-analysis indicate that elevated preoperative PLR is predictive of poor OS, CSS/DSS and RFS/DFS in urothelial carcinoma patients. However, the pooled HRs were close to 1, and there were several limitations to our study. Therefore, the prognostic value of preoperative PLR should be cautiously interpreted when considering urothelial carcinomas in clinical practice. It is noteworthy that no significant correlation was found between high preoperative PLR and advanced pathological tumor stage (T ≥ 2), presence of tumor necrosis or high tumor grade (G ≥ 3). In view of the aforementioned limitations, additional more multicenter and large scale studies will be needed to validate preoperative PLR as an independent risk factor in the management of urothelial carcinoma patients.

## MATERIALS AND METHODS

### Literature search

A comprehensive literature search of Pubmed, Embase and Web of science, Wanfang and Chinese National Knowledge Infrastructure up to April 27th, 2017, was performed to assess the prognostic role of PLR in the patients with urothelial carcinoma. We performed this systematic review and meta-analysis according to the Preferred Reporting Items for Systematic Reviews and Meta-Analyses statement (PRISMA) [29], and the PRISMA checklist was shown in Supplementary Table 3. The search query combined the keywords as follows: (bladder cancer OR bladder carcinoma OR urothelium cancer OR urothelial carcinoma OR ureteral carcinoma OR ureteral cancer OR renal pelvic carcinoma OR renal pelvic cancer) AND (PLR OR platelet to lymphocyte ratio OR platelet lymphocyte ratio OR platelet-lymphocyte ratio). Bibliographies of related studies were also checked for potential inclusions.

### Inclusion and exclusion criteria

According to the PICO criteria [30], the included studies should satisfy all the following items: (1) all the included patients were diagnosed as urothelial carcinoma in urinary tract; (2) the PLR was collected preoperatively and calculated as the ratio of absolute platelet count to lymphocyte count; (3) all the included studies reported cut-off values for comparison; (4) sufficient data were reported for extraction of HRs and related 95% CIs with prognostic endpoints. Otherwise, the studies were excluded according to the following criteria: (1) duplicates detected manually or by Mendeley software; (2) editorials, conference abstracts, review articles, related studies without sufficient data; (3) studies comprising < 10 patients; (4) HRs and 95% CIs were derived from the overlapping patient or analyzed with continuous variable for PLR; (5) studies were not published in English.

### Data extraction and evaluation of the included studies

The related data were extracted with a predefined form, the details were listed in the following: first author’s name, year of publication, study design, cut-off value, prognostic endpoints (OS, CSS/DSS or RFS/DFS). HRs and their 95% CIs were directly extracted from multivariate or univariate analyses. The raw data reported the association between PLR and the main pathological results in the included studies was also collected for further analysis. The main pathological results included tumor stage, tumor grade, tumor necrosis.

The methodological quality of each study was evaluated with the tool reported by Steels E [31]. The tool included 4 sub-items, and the total scores ranged from 0 to 44. The global scores are expressed as percentages, with a higher scores indicating a better methodological quality [31, 32]. Both data extraction and methodological evaluation were perfomed independently by two reviewers, and any discrepancies were addressed by the discussion with a third reviewer.

### Data analysis

The pooled HRs and 95% CIs were conducted by the software Stata version 12.0, and sub-group analysis were performed according to different cancer types (UCB, UUTUC). The correlations between PLR and pathological results were calculated as odds ratios (ORs) and its 95% CIs with SPSS 19.0 software. Heterogeneity of the included studies was shown with Chi-squared, P_h_ value and I-square (I^2^). P_h_ > 0.10 or I^2^ value < 50% was defined as no significant heterogeneity [32]. We preferred to take the fixed-effects model to analyse the pooled HRs if no significant heterogeneity was found. Otherwise, we chose random-effects model to analyse the data.The results always predicted poor prognosis if the lower 95% CIs was greater than 1. We preferred to extract the HRs derived from multivariate analysis if both univariate and multivariate Cox regression analyses were performed in the same study. Both Begg’s funnel plot and Egger’s test were performed to evaluate the publication bias among the included studies. “Trim and fill” method was conducted to explore hypothesized missing studies and adjust the pooled results [33–35]. *P* > 0.05 was defined as no significant asymmetry.

## SUPPLEMENTARY MATERIALS FIGURES AND TABLES




