# Supplementary material for: Systematic review and meta-analysis of the prognostic value of preoperative platelet-to-lymphocyte ratio in patients with urothelial carcinoma
Source: Oncotarget. 2017 Sep 22;8(53):91694–702. doi: 10.18632/oncotarget.21162 (PMC5710958; doi:10.18632/oncotarget.21162)
Supplement: Supplementary file 1 [file oncotarget-08-91694-s001.pdf]

Systematic review and meta-analysis of the prognostic value of preoperative platelet-to-lymphocyte ratio in patients with urothelial carcinoma

SUPPLEMENTARY MATERIALS

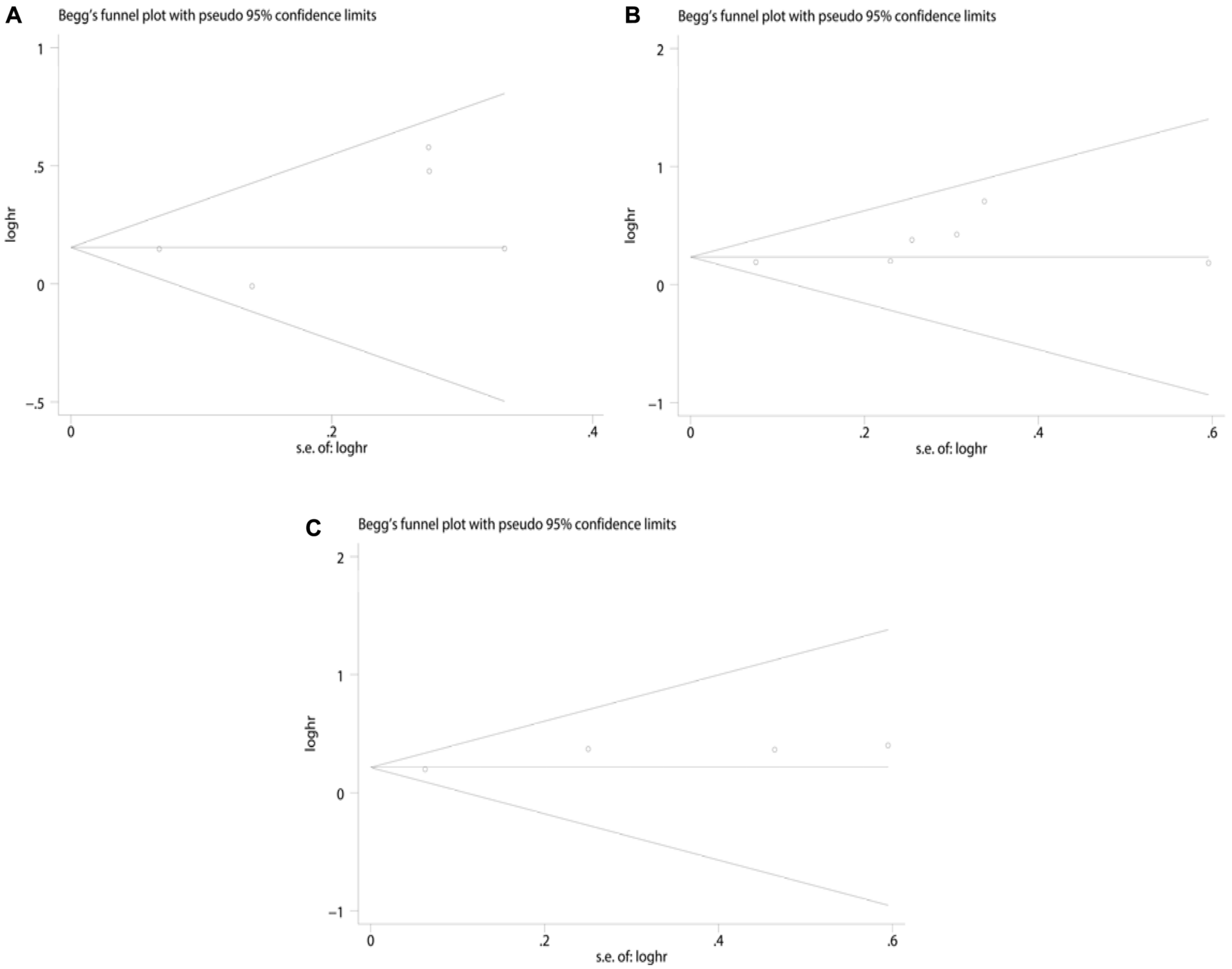

Supplementary Figure 1: Begg's funnel plot evaluating the potential publication bias among the included studies. (**A**) PLR with OS (*P* = 0.806); (**B**). PLR with DSS/CSS (*P* = 0.13); (**C**). PLR with RFS/DFS (*P* = 1.00).

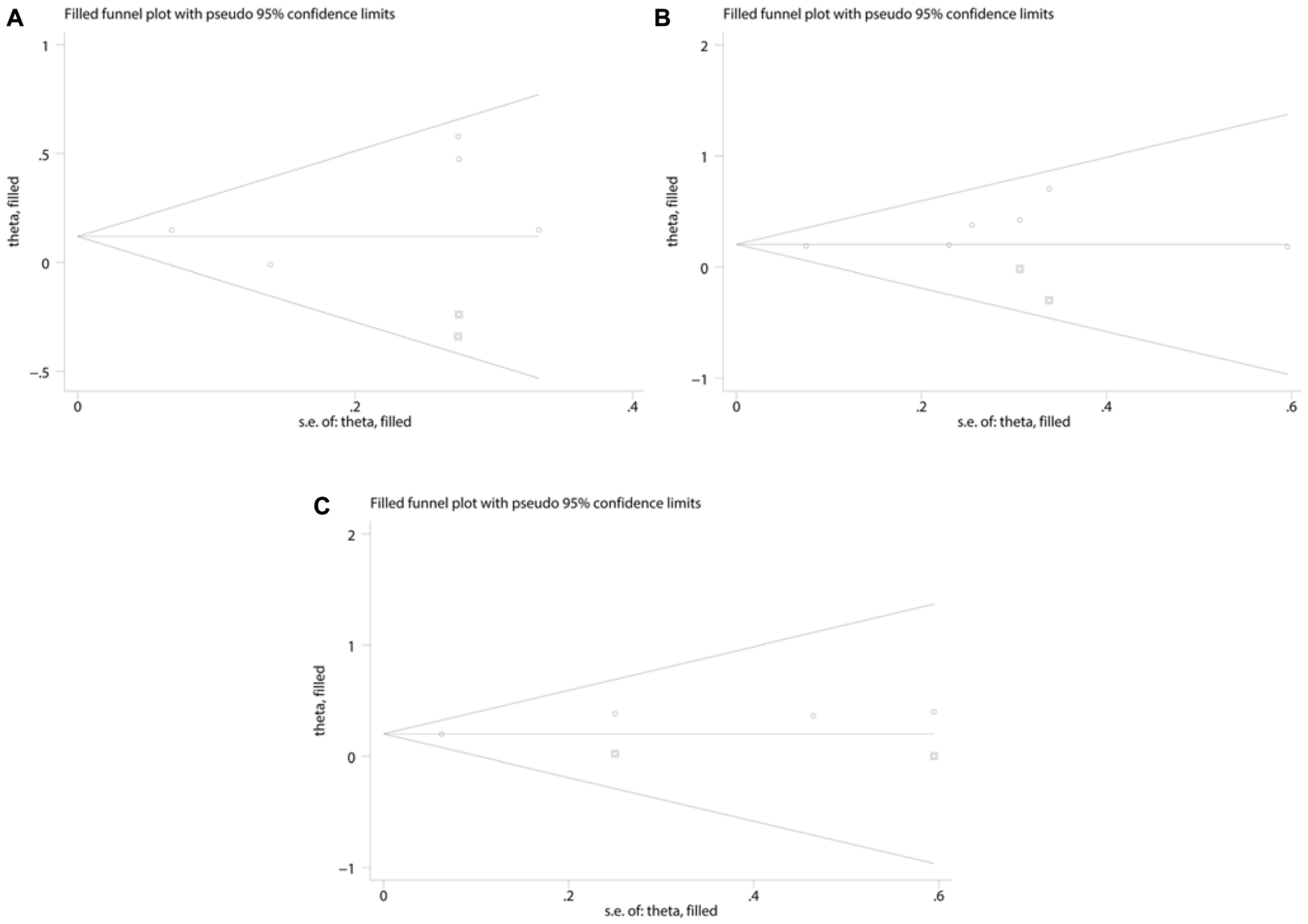

Supplementary Figure 2: "Trim and fill" analyses: (**A**) PLR with OS (HR = 1.13, 95%CI = 1.01–1.25, *P* = 0.03) (Ph = 0.12, fixed effect model); (**B**). PLR with DSS/CSS (HR = 1.23, 95%CI = 1.09–1.39, *P* < 0.01) (Ph = 0.55, Fixed effect model); (**C**). PLR with RFS/DFS (HR = 1.22, 95%CI = 1.09–1.37, *P* < 0.01) (Ph = 0.93, Fixed effect model.

Supplementary Table 1: Pooled hazard ratios(HRs) for OS according to subgroup analyses

Subgroup	Number of studies	Number of patients	Effect models	HR (95%CI)	Heterogeity	
					I^2 (%)	*P*
Cut-off value for PLR						
≥ 150	2	661	Fixed	1.01 (0.75–1.27)	0	0.70
< 150	2	1675	Fixed	1.69 (1.01–2.36)	0	0.80
Per 100 unit	1	418	Fixed	1.16 (1.00–1.31)	-	-
Number of patients						
> 400	3	2450	Fixed	1.13 (1.00–1.26)	0	0.35
< 400	2	304	Fixed	1.41 (0.78–2.04)	9.4	0.33
Ethnicity						
Asian	3	2156	Fixed	1.05 (0.80–1.30)	0	0.43
Caucasian	2	598	Fixed	1.17 (1.02–1.33)	30.5	0.23
Cancer types						
UCB	3	2093	Fixed	1.12 (0.99–1.25)	0	0.57
UUTUC	2	661	Fixed	1.69 (1.01–2.36)	0	0.80
Overall	5	2754	Fixed	1.14 (1.01–1.27)	0	0.43

Supplementary Table 2: Pooled hazard ratios (HRs) for DSS/CSS according to subgroup analyses

Subgroup	Number of cohorts	Number of patients	Effect models	HR (95%CI)	Heterogeity	
					I^2 (%)	*P*
Number of patients						
> 400	3	2450	Fixed	1.22 (1.05–1.39)	0	0.82
< 400	3	457	Fixed	1.53 (0.91–2.16)	0	0.73
Ethnicity						
Asian	4	2309	Fixed	1.34 (0.94–1.74)	0	0.93
Caucasian	2	598	Fixed	1.22 (1.04–1.40)	17.40	0.27
Cut–off value for PLR						
≥ 150	4	938	Fixed	1.53 (1.01–2.06)	0	0.89
< 150	1	1551	Fixed	1.21 (1.03–1.39)	–	–
Per 100 unit	1	418	Fixed	1.22 (0.65–1.79)	–	–
Cancer types						
UCB	2	1969	Fixed	1.21 (1.04–1.38)	0	0.97
UUTUC	4	938	Fixed	1.53 (1.01–2.06)	0	0.89
Overall	6	2907	Fixed	1.24 (1.08–1.40)	0	0.86

Supplementary Table 3: PRISMA 2009 Checklist. See Supplementary_Table_3
